# Rat locomotor spinal circuits in vitro are activated by electrical stimulation with noisy waveforms sampled from human gait

**DOI:** 10.1002/phy2.25

**Published:** 2013-07-08

**Authors:** Francesco Dose, Rachele Menosso, Giuliano Taccola

**Affiliations:** 1 Neuroscience Department, International School for Advanced Studies (SISSA)via Bonomea 265, Trieste, Italy; 2 SPINAL (Spinal Person Injury Neurorehabilitation Applied Laboratory), Istituto di Medicina Fisica e Riabilitazione (IMFR)via Gervasutta 48, Udine, Italy; 3 IMFR, ASS4 Medio Friulivia Gervasutta 48, Udine, Italy

**Keywords:** CPG, EMGs, spinal cord

## Abstract

Noisy waveforms, sampled from an episode of fictive locomotion (FL) and delivered to a dorsal root (DR), are a novel electrical stimulating protocol demonstrated as the most effective for generating the locomotor rhythm in the rat isolated spinal cord. The present study explored if stimulating protocols constructed by sampling real human locomotion could be equally efficient to activate these locomotor networks in vitro. This approach may extend the range of usable stimulation protocols and provide a wide *palette* of noisy waveforms for this purpose. To this end, recorded electromyogram (EMG) from leg muscles of walking adult volunteers provided a protocol named ReaL*i*stim (Real Locomotion-*induced* stimulation) that applied to a single DR successfully activated FL. The smoothed kinematic profile of the same gait failed to do so like nonphasic noisy patterns derived from standing and isometric contraction. Power spectrum analysis showed distinctive low-frequency domains in ReaL*i*stim, along with the high-frequency background noise. The current study indicates that limb EMG signals (recorded during human locomotion) applied to DR of the rat spinal cord are more effective than EMG traces taken during standing or isometric contraction of the same muscles to activate locomotor networks. Finally, EMGs recorded during various human motor tasks demonstrated that noisy waves of the same periodicity as ReaL*i*stim, could efficiently activate the in vitro central pattern generator (CPG), regardless of the motor task from which they had been sampled. These data outline new strategies to optimize functional stimulation of spinal networks after injury.

## Introduction

One important goal for spinal network rehabilitation is the possibility to activate locomotor patterns with electrical stimuli applied to afferent inputs (Harkema et al. 2011). This is particularly attractive as a tool to recover, at least in part, locomotor activity after spinal cord injury. In the attempt to optimize the parameters for such a stimulation using as a test model the in vitro spinal cord preparation, we recently discovered a new stimulating protocol, named FL*i*stim (Fictive Locomotion-*induced* stimulation) based on high-frequency sampling of FL records from a ventral root (VR) of an isolated neonatal rat spinal cord and delivering it to a single dorsal root (DR) of the same preparation (Taccola 2011; Dose and Taccola 2012). This special stimulation pattern, even when applied at amplitude lower than the one required by standard square pulses, was able to induce locomotor-like oscillations of longer duration and with a greater number of cycles than hitherto described (Taccola 2011). The specific recruitment of the locomotor central pattern generator (CPG) made by FL*i*stim is confirmed by its ability to synergize the FL induced by NMDA (N-Methyl-D-aspartate) + 5-HT (5-hydroxytryptamine; Dose and Taccola 2012). Although the precise mechanisms through which FL*i*stim can activate locomotor CPG remain unclear, its intrinsic noise turns out to be a crucial feature (Taccola 2011). The results so far were exclusively obtained using FL patterns sampled from neonatal rat spinal cords. We wondered if collecting records of limb muscle activity during real locomotion from healthy human volunteers might also have the ability to induce FL in the isolated rat spinal cord. This seems to be a desirable goal because the characteristics of human and rodent locomotor patterns, although similar, are not identical. Furthermore, using human electromyogram (EMG) data also allows DR stimulation with noisy, nonphasic traces (obtained during isometric or postural contraction of antigravity muscles) to assess the relative role of noise in the CPG activation.

Furthermore, detailed analysis of the EMG recorded from human leg muscles has allowed identifying distinct activation profiles during the execution of a specific motor task (Raasch and Zajac 1999; Bizzi et al. 2008; Wakeling and Horn 2009). Thus, we can hypothesize that stimulating protocols obtained from EMGs sampled during several motor tasks may activate, more or less efficiently, the in vitro CPG. To this aim, EMGs referred to distinct rhythmic movements, such as pedaling, hopping, or jumping, were recorded from volunteers, digitized and applied to a single DR to assess their impact on the locomotor CPG in vitro. Finally, stimulating patterns obtained from the EMG captured from repetitive flexions of the ankle joint were used to evaluate whether noisy waves sampled from monoarticular rhythmic oscillations were per se sufficient to activate the in vitro CPG.

## Methods

### Electrophysiological recordings

All procedures were conducted in accordance with the guidelines of the National Institutes of Health and the Italian Act Decreto Legislativo 27/1/92 n. 116 (implementing the European Community directives n. 86/609 and 93/88) and under the authorization of the Italian Ministry of Health. All efforts were made to reduce the number of animals and their suffering.

Experiments were performed on spinal cord preparations after isolation from neonatal rats (P0-P4), as previously reported (Taccola et al. 2004). Briefly, spinal cords were sectioned from the midthoracic region to the *cauda equina*, maintained at a constant room temperature of 22°C and continuously superfused (5 mL/min) with oxygenated (95% O_2_; 5% CO_2_) Krebs solution of the following composition (in mmol/L): 113 NaCl, 4.5 KCl, 1 MgCl_2_7H_2_O, 2 CaCl_2_, 1 NaH_2_PO_4_, 25 NaHCO_3_, and 11 glucose, pH 7.4. VR recordings in DC mode were taken from L2 VRs, which contain axons from motoneurons that innervate mainly hindlimb flexor muscles, and from L5 VRs containing axons which drive primarily hindlimb extensor muscles (Kiehn and Kjaerulff 1996). The alternation of discharges between flexor and extensor motor pools and between left (l) and right (r) sides of the cord represents the hallmark of FL (Juvin et al. 2007).

### Parameters of spinal network activity

Electrical stimuli were delivered, using bipolar suction electrodes, in order to evoke single VR responses. Stimuli were considered as threshold (Th) according to their ability to elicit fast synaptic responses from the homologous VR (see Marchetti et al. 2001). In response to DR stimulation with repetitive stimulating patterns, epochs of FL arise over a background of cumulative depolarization. FL cycles were analyzed for their periodicity (time between the onset of two consecutive cycles of oscillatory activity) and regularity, expressed by the coefficient of period variation (CV; displayed as standard deviation [SD] mean^−1^). The correlation among signals arising from pairs of VRs was expressed by the cross-correlogram function (CCF), obtained using Clampfit® 10.3 software (Molecular Devices LLC, CA). While a CCF greater than −0.5 indicated that two VRs were synchronous, a CCF less than −0.5 showed full alternation (Ryckebusch and Laurent 1994; Taccola et al. 2010).

The power spectrum for stimulating patterns was obtained through Clampfit® 10.3 software (Molecular Devices, LLC, CA).

### Designing the ReaL*i*stim

EMG recordings from several muscles were obtained during various motor tasks carried out by two healthy volunteers. Epochs (60 sec) of EMG traces were promptly processed for off-line analysis (Clampfit® 10.3 software; Molecular Devices) and records from one muscle were randomly selected for use. The sampled trace was imported into a spreadsheet of Origin® 9 (OriginLab, North Hampton, MA), where the *x*-axis considered each sampling time for the epoch duration and the *y*-axis was used for the corresponding current amplitude. The two columns of values were then exported (as an ASCII text file) to a programmable stimulation device (STG® 2004; Multi Channel Systems, Reutlingen, Germany). The stimulating protocol resulting from this procedure was termed ReaL*i*stim (Real Locomotion-*induced* stimulation) and was applied to a DR.

### Subjects

Two healthy right leg dominant subjects (female, 31 years old, 62 kg, 1.74 m; male, 43 years old, 76 kg, 1.77 m) volunteered for the experiments. Human recordings were performed by specialized health care professionals in the SPINAL Clinical lab at Istituto di Medicina Fisica e Riabilitazione (Udine, Italia) and in the Gait & Motion Analysis Laboratory at Sol et salus hospital (Torre Pedrera, Rimini, Italy). The study conformed to the Declaration of Helsinki and the written informed consent was obtained from participants according to the clinical protocols established by the Istituto di Medicina Fisica e Riabilitazione (Udine, Italia).

### EMG recordings

Spectrum profiles of EMG intensities were continuously obtained for 60 sec during different motor tasks, namely standing, overground walking at a self selected speed, incremental squats at 30°, 60°, and 90° of knee flexion, hopping, two legged or one legged jumping, and pedaling on an Ergoselect® 100K bicycle ergometer (Ergoline GmbH, Deutschland) at the frequency of 60 rpm and power output of 120 W.

EMG recordings from rhythmic oscillations of the right ankle joint, featuring a cycle period approximating that for walking and cycling, were also taken from a subject lying supine with one foot supported by a researcher.

During the performance of these exercises, subjects were instructed to generate stepping, jumping, or ankle flexions at the same frequency using an auditory metronome.

Briefly, EMG Ag/AgCl surface electrodes (10 mm diameter, 21 mm interelectrode distance) were positioned on the right leg and connected to TELEMG® system (BTS, Milano, Italia). Guidelines provided by the European Project SENIAM (Surface EMG for Non Invasive Assessment of Muscles, 1996–1999; Hermens et al. 2000) were followed in positioning electrodes. EMG signals were simultaneously recorded from the tibialis anterior (TA), medial gastrocnemius (GM), vastus medialis (VM), rectus femoris (RF), and vastus lateralis (VL). The EMG signals were band-pass filtered, with cut-off frequencies from 5 to 200 Hz, amplified 1000× and then sampled at 500 Hz.

### Analysis of EMG signals and gait

As a control to define the role of intrinsic variability of the stimulating pattern in effectively activating the CPG, we compared alternating oscillations induced by noisy waves recorded from limb muscles with the effects elicited by simultaneously derived kinematic patterns (from limb markers) that were smooth sinusoids. Kinematic profiles were obtained with the Elite® 2000 system (BTS, Milano, Italia) consisting of six infrared cameras (positioned 4.5 m along the progression line of the subjects) operating at a sampling frequency of 50 Hz (Ferrigno and Pedotti 1985). After three-dimensional calibration, the spatial accuracy of the system was greater than 1.5 mm. Infrared reflective marks were positioned in correspondence to the lateral condyle of the knee (knee), the lateral malleolus (mall), between the lateral condyle of the knee and the lateral malleolus in the midpoint (bar), the heel, and the 5th metatarsal (met; Davis et al. 1991). Subjects were asked to walk barefoot as naturally as possible, looking straightforward. During the acquisition of kinematic data, the activity of soleus muscle was simultaneously recorded as described above.

### Statistical analysis

All data are reported as mean ± SD, where n indicates the number of spinal cord preparations. After distinguishing between parametric or nonparametric data, using a normality test, all parametric values were analyzed with Student's *t*-test (paired or unpaired) to compare two groups of data or with analysis of variance (ANOVA) for more than two groups. For nonparametric values, Mann–Whitney test was used for two groups, while, for multiple comparisons, ANOVA on Ranks was first applied, followed by a post hoc test (Dunnett's method). Statistical analysis was performed using SigmaStat 3.5 software (Systat Software Inc, IL). Results were considered significant when *P* < 0.05.

## Results

### Noisy waves obtained from EMG recordings of the lower limb during human locomotion activate the in vitro CPG

**Figure 1:**
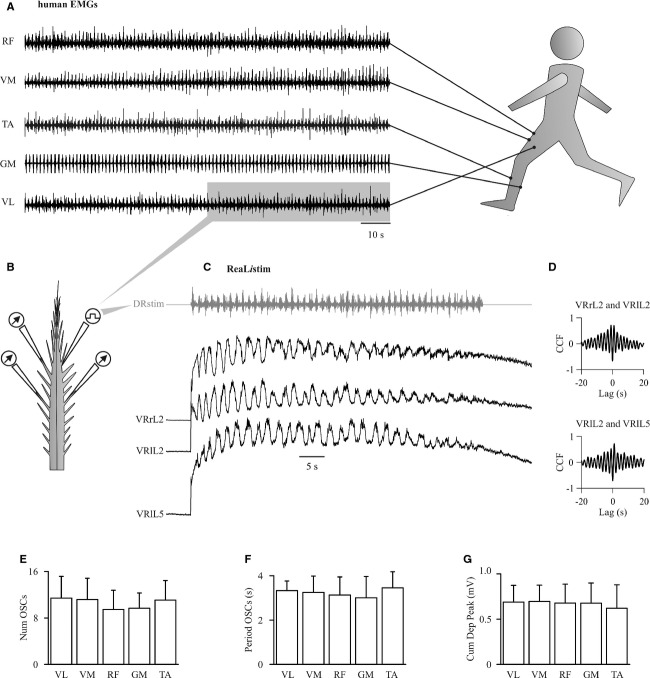
EMG recordings during human locomotion provide noisy waveforms able to activate the in vitro CPG. In A, EMGs are simultaneously recorded from five muscles of the right leg during real locomotion in an adult volunteer walking overground at normal speed. The superficial electrodes are positioned as indicated by the black lines in the cartoon (right). A segment of 60 sec duration is extracted through offset analysis from the VL EMG (shaded box) and used for designing the ReaL*i*stim protocol, later delivered to the DRrL6 of a neonatal rat isolated spinal cord (intensity = 0.54 Th), while the motor response is continuously monitored through recording suction electrodes from VRs L2 and L5 (B). During electrical stimulation with ReaL*i*stim, VRs are depolarized with a superimposed episode of locomotor-like oscillations (C), as confirmed by the negative peaks centered at zero lag value in the cross-correlogram functions in D. In E, F, and G, histograms show, respectively, that the mean number and the mean period of FL oscillations, as well as the mean cumulative depolarization amplitude, do not statistically change, even in response to delivery of noisy waveforms obtained from different leg muscles (*n* = 7; *P* = 0.723 for E; *P* = 0.740 for F; *P* = 0.967 for G).

We aimed at assessing whether electrical stimulation with noisy waveforms corresponding to locomotor patterns of an adult volunteer was able to trigger the CPG of the in vitro neonatal rat spinal cord. Thus, we first recorded EMGs from five muscles in the lower limb (RF, VM, TA, GM, and VL; Fig. 1A) of a volunteer walking at a freely chosen stride frequency (average speed = 1.01 m/sec). For each EMG trace, a 60 sec segment was randomly sampled in order to design the stimulation protocol, that we named ReaL*i*stim, which, in this example, was characterized by noisy waves with average 1.25 sec period. Figure 1A exemplifies how the noisy traces obtained from the VL muscle (shaded box), when delivered (intensity = 0.54 Th) to rL6 DR of the isolated rat spinal cord (Fig. 1B), evoked cumulative VR depolarization (1.17 mV) with superimposed 28 oscillating cycles (2.06 ± 0.41 sec period and 0.20 CV; Fig. 1C). Oscillations between L2 and L5 VRs on the two sides of the spinal cord were alternated, as confirmed by the value of the cross-correlograms illustrated in Figure 1D (CCF homolateral = −0.71; CCF homosegmental = −0.66), indicating, therefore, their characteristic FL property.

EMG recordings obtained in three sessions of locomotion (average speed of 1.11 ± 0.07 m/sec) provided ReaL*i*stims (period = 1.12 ± 0.11 sec for 60 sec epoch), which induced, in 11 preparations, cumulative VR depolarization of 0.71 ± 0.33 mV with a superimposed episode of FL (52.15 ± 7.08 sec long, 21 ± 4 locomotor cycles of 2.73 ± 0.65 sec period and 0.28 ± 0.11 CV). In six experiments, ReaL*i*stims, simultaneously sampled from flexor and extensor muscles of leg and calf, and delivered in sequence to a single DR of the isolated rat spinal cord, were equally capable of evoking locomotor-like responses of similar duration, period, and cumulative depolarization (Fig. 1E–G).

### Different responses of the in vitro CPG to electrical stimulation with EMGs or kinematic profiles

Stimulating patterns composed of noisy or smooth sinusoidal waves of identical main frequency were obtained by simultaneously recording, during the same session of human locomotion, the activity of the soleus muscle, and the variations in the joint profile of the heel, on the *y*-axis. The two traces were imported into the programmable stimulator and corrected to obtain waveform traces of the same maximal amplitude. Hence, the two stimulating patterns (ReaL*i*stims and kinematic profiles) provided either a noisy baseline or a smooth baseline.

**Figure 2:**
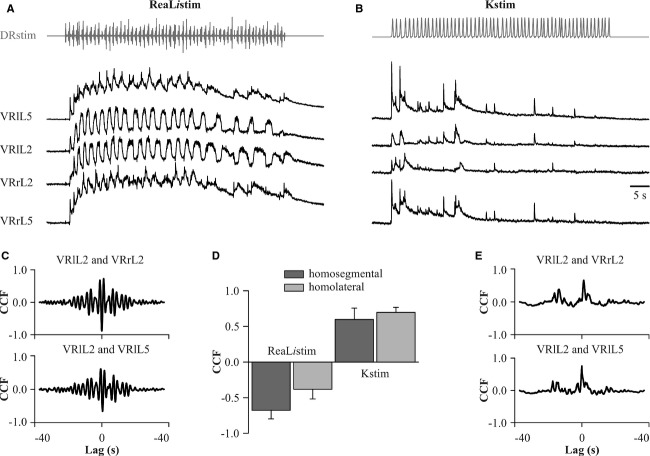
Kstim, unlike ReaL*i*stim, does not trigger FL. In A, ReaL*i*stim, designed from right soleus EMG (intensity = 0.33 Th), when applied to DRrS1, evokes an episode of FL from VRs L2 and L5 on both side of the spinal cord. In the same experiment, delivery of Kstim, sampled from the kinematic profile of right heel (intensity = 0.33 Th), generates only a slight cumulative depolarization with sporadic electrical discharges (B). In C, the cross-correlogram analysis performed for the traces shown in A reports negative values, representing the double alternation among pairs of homosegmental L2 (top) and homolateral L2 and L5 (bottom) VRs. Conversely, in E, the cross-correlogram analysis for traces in B shows positive peaks, describing full synchrony among discharges recorded from homosegmental (top) and homolateral (bottom) VRs. In D, histograms summarize the mean CCF values obtained for homosegmental (dark gray) and homolateral (light gray) VRs in response to stimulation of the same cords with ReaL*i*stim (right) and Kstim (left), respectively. While ReaL*i*stim always evokes alternating oscillations, Kstim generates only synchronous events (*n* = 5).

Figure 2A shows that stimulation of the DRrS1 of the isolated spinal cord with ReaL*i*stim (intensity = 0.33 Th) induced cumulative depolarization (0.46 mV) with FL of 57.31 sec duration with 22 cycles (period and CV of 2.73 ± 1.03 sec and 0.38, respectively). Conversely, on the same preparation, electrical stimulation of the same DR with the protocol obtained from the kinematic profile of the heel (intensity = 0.33 Th) induced very small cumulative depolarization (0.08 mV), with a series of synchronous discharges among the four VRs. The cross-correlogram analysis (Fig. 2C and E) confirms that ReaL*i*stim evoked FL with alternating oscillations among homosegmental (lL2 and rL2, CCF = −0.88) and homolateral (lL2 and lL5, CCF = −0.66) VRs, while a sinusoidal stimulation obtained from the heel kinematic profile evoked only synchronous oscillations both at homosegmental (VRlL2 and VRrL2, CCF = 0.65) and homolateral (VRlL2 and VRlL5, CCF = 0.75) levels.

These results were confirmed in five preparations (Fig. 2D) with mean CCF of −0.68 ± 0.12 for homosegmental VRs and of −0.38 ± 0.14 for homolateral VRs during ReaL*i*stim. In the same preparations, delivery of waves obtained from the kinematic analysis of the heel evoked synchronous discharges (homosegmental CCF = 0.60 ± 0.16 and homolateral CCF = 0.70 ± 0.07) only.

**Figure 3:**
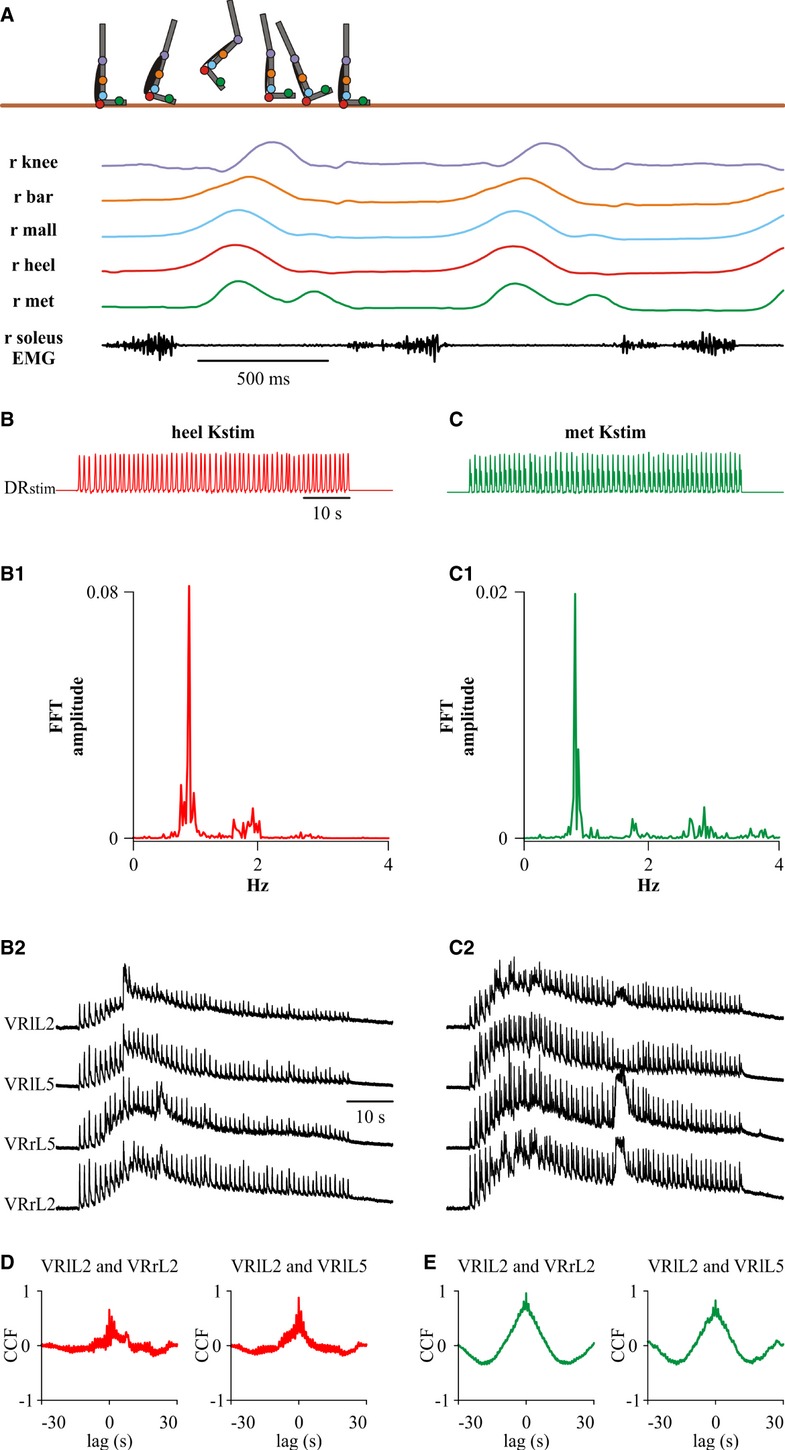
Kstims sampled from different tracks of the lower limb are ineffective in eliciting FL. The kinematic analysis of locomotion is performed on several tracks of the right leg (knee, bar, mall, heel, and met), as reconstructed for consecutive phases of the first step in A. The kinematic profiles are indicated in gray scale and synchronized with the soleus muscle EMG. Segments of 60 sec duration are sampled from heel and met profiles to produce the stimulating protocols heel Kstim (B) and met Kstim (C), for which power spectra are obtained (B_1_ and C_1_, respectively). Heel Kstim applied to DRrS4 (intensity = 0.33 Th) evokes a small cumulative depolarization accompanied by single reflex responses, corresponding to stimulating pattern peaks, and by the sporadic appearance of bursts (B_2_). Analogously, met Kstim (intensity = 0.33 Th) depolarizes VRs, with single reflex responses and sporadic bursts that are synchronous among all VRs (C_2_). The cross-correlogram analysis from pairs of homosegmental (left) and homolateral (right) VRs quantifies the synchrony among discharges elicited by Kstims (D for heel Kstim and E for met Kstim).

We tested whether the inability to activate the in vitro FL with Kstim was due to the absence of noise in kinematic profiles. For this reason, numerous kinematic patterns were simultaneously sampled, on the *y*-axis, from different track positions in the lower limb during the same locomotor session. As schematized in Figure 3A, in correspondence to gait phases exemplified as a stick diagram, kinematic traces from different tracks of the lower limb were recorded for the first two steps of a locomotor session. These traces were synchronized with the EMG recorded from the right soleus (bottom record in Fig. 3A). The metatarsal trace presented the most different profile from the one from the heel as it comprises a second peak in coincidence with ankle flexion. Fourier analysis (Fig. 3B_1_–C_1_) confirms that both traces had a main peak at 0.9 Hz and a second component at 1.8 Hz, while the metatarsal trace shows a further component at 2.9 Hz (Fig. 3C).

As illustrated in Figure 3B_2_ and C_2_, we subsequently delivered, to the same DR in the same preparation, first, a smoothed wave obtained from the heel trace and, then, the one recorded from the metatarsus, both at the same maximum amplitude. Responses from VRs (see Fig. 3B_2_– and C_2_) indicate that, in both cases, the stimulation induced similar cumulative depolarization (0.35 μV for the heel and 0.41 μV for the metatarsus), followed by baseline repolarization despite continuous stimulation. Reflex discharges were observed during each stimulating cycle plus sporadic, slow bursts. The cross-correlogram analysis (Fig. 3D and E) confirms events synchronicity among homosegmental (heel CCF = 0.66; met CCF = 0.83) and homolateral (heel CCF = 0.88; met CCF = 0.96) VRs. The same observations were obtained from three spinal cords. These results indicate that, unlike ReaL*i*stim, Kstim could not evoke FL, suggesting that the noise contained in the stimulating traces sampled from the motor output of a human individual was a crucial characteristic for activating the in vitro CPG.

### Noisy traces obtained during isometric contractions or static posture do not induce FL

**Figure 4:**
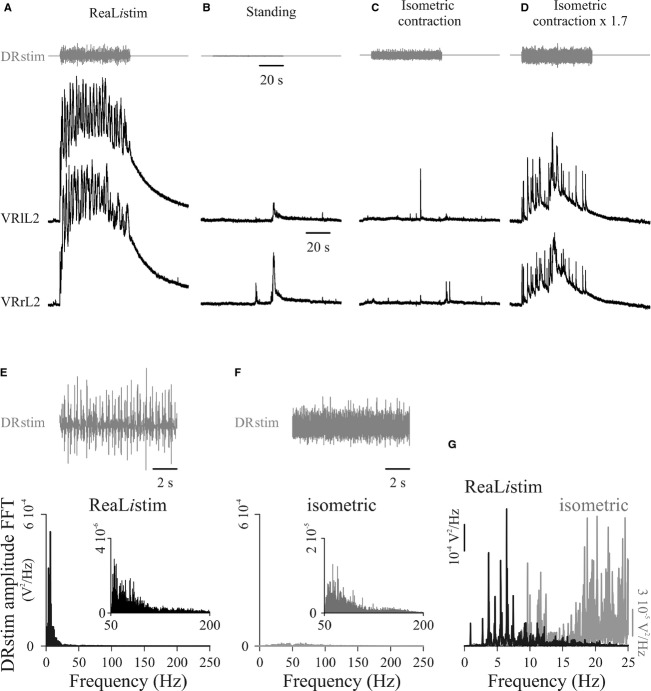
Human EMGs sampled during standing and isometric contraction fail in inducing FL. In A, ReaL*i*stim is designed from rVM EMG recording and applied to DRlL7 (intensity = 0.15 Th) to elicit an epoch of alternating oscillations from L2 VRs. In the same experiments, delivery of the EMGs recorded from rVM during standing static posture (B, intensity = 0.01 Th) or isometric muscle contraction (C, intensity = 0.09 Th) does not depolarize VRs, although elicits few uncorrelated discharges. Finally, when the peaks of the EMG in C are artificially adjusted to the same maximum amplitude of ReaL*i*stim in A, the resulting stimulating protocol can generate a small cumulative depolarization with synchronous events (D). In E, a segment of the same ReaL*i*stim shown in A is displayed at a faster time base scale, while the power spectrum of the entire stimulating pattern (60 sec) is reported below. An analogous Fourier analysis is performed also for isometric EMGs (F, in top panel see a sample episode of the DRstim in C, at a faster time base scale). Note in the inserts the magnification of spectra in the high-frequency regions. The power spectra of the two protocols are superimposed in G (black trace, ReaL*i*stim; gray trace, isometric contraction), emphasizing that, among the noisy waveforms sampled from EMGs, only ReaL*i*stim presented a series of components in the low-frequency domain.

To further clarify the characteristics of ReaL*i*stim responsible for the activation of the in vitro CPG, we have explored whether the EMG phasic patterns, used to design ReaL*i*stim, were a crucial characteristic. Thus, we compared stimulation using ReaL*i*stim (see top record in Fig. 4A) with noisy traces derived from the tonic activation of the same muscle, either during the antigravity contraction for standing (top trace in Fig. 4B) or during an isometric contraction task (top record in Fig. 4C and D).

As demonstrated by Figure 4A, 60 sec stimulation with ReaL*i*stim (VM muscle) evoked (intensity = 0.15 Th) cumulative depolarization (0.80 mV) with a longlasting episode of FL (57.77 sec, 23 oscillations, period and CV of 2.62 ± 0.51 sec and 0.20, respectively). Oscillations alternated among homosegmental VRs, as confirmed by the cross-correlogram analysis (homosegmental CCF = −0.50; not shown).

On the other hand, stimulation with EMG traces sampled during static posture or isometric contraction did not evoke cumulative depolarization, but only sporadic tonic discharges (Fig. 4B and C). Even when increasing the amplitude of the EMG trace from the isometric contraction to that of the ReaL*i*stim one, no alternating oscillations appeared, although the stimulating protocol was able to induce cumulative VR depolarization (0.31 mV; Fig. 4D).

Analogous observations were replicated with six preparations, in which ReaL*i*stim induced an average cumulative depolarization of 0.66 ± 0.29 mV, superimposed by FL of 53.65 ± 3.98 sec duration with 20 ± 2 alternating cycles of period and CV 2.90 ± 0.36 sec and 0.29 ± 0.11, respectively. In the same spinal cords, in response to stimulation with EMGs recorded during standing posture or isometric contractions, we did not observe any depolarization or appearance of locomotor oscillations. Even stimulation with the EMG traces of an isometric contraction with amplitude brought to ReaL*i*stim values, did not induce any locomotor cycles, despite an average cumulative depolarization of 0.36 ± 0.13 mV. These findings suggest that the basic intrinsic noise of the human EMG was unable to elicit FL.

To look for discrete frequency domains within the noisy waveforms necessary for activate FL, we analyzed the power spectra of the ReaL*i*stim record (see, in Fig. 4E, example at high gain and faster time base taken from the record shown in A) and isometric contraction trace (see, in Fig. 4F, example at high gain and faster time base taken from the record shown in C). While the power spectrum of ReaL*i*stim (Fig. 4E, bottom) revealed principal components clustered at the low frequency, on the other hand, the spectrum of isometric contraction lacked these peaks and contained only small components at higher frequency (Fig. 4F, bottom). The insets to Figure 4E and F show that, in the high-frequency domain, a similar pattern for both protocols (albeit smaller in amplitude for ReaL*i*stim) was present. Figure 4G indicates that, after alignment of both traces in the frequency range below 25 Hz, ReaL*i*stim (black trace) was characterized by a series of components between 0 to 10 Hz, whereas the isometric contraction stimulus (gray trace) lacked any elements. On the contrary, from 15 to 25 Hz, only the isometric contraction profile was observed (Fig. 4G).

### Effect of stimuli sampled from human EMGs during nonlocomotor rhythmic activity

We next tested whether stimulation with noisy waveforms from EMG recordings of the right GM during the execution of nonlocomotor rhythmic activities could activate the in vitro CPG. Thus, we sampled 60 sec EMG records taken while one volunteer was pedaling or hopping.

**Figure 5:**
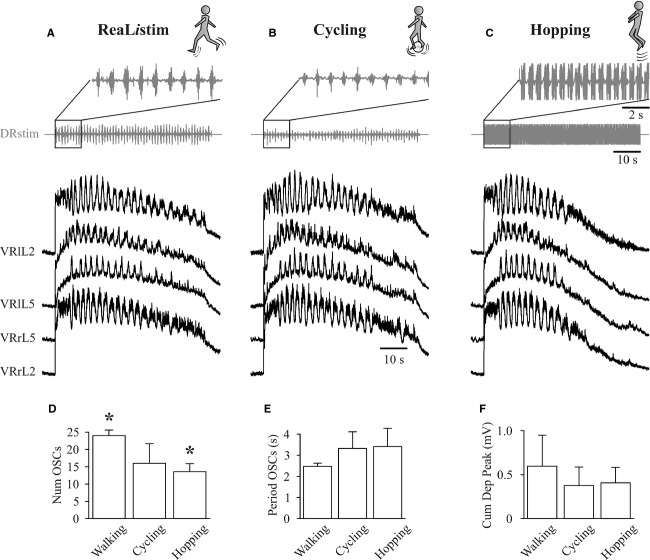
Stimulating protocols obtained from human EMGs during walking, cycling, and hopping induce a different number of FL cycles. In A, ReaL*i*stim is designed from the EMG recording (duration 60 sec; in top panel is reported a faster sample trace) of rGM during locomotion. The stimulating pattern is applied to DRlL6 (intensity = 0.50 Th), evoking an episode of FL from L2 and L5 VRs on both side of the cord. A FL episode of duration similar is generated by a stimulating protocol (duration = 60 sec; intensity = 0.40 Th), sampled from the rGM EMG recording while the volunteer is pedaling (B, in top panel is reported a faster sample trace). Contrarily, by applying a rGM EMG trace (duration = 60 sec; intensity = 0.50 Th; in top panel is reported a faster sample trace) recorded during hopping a shorter episode of FL is obtained. Note that A, B, and C are referred to the same preparation. Histograms in D, E, and F summarize the mean value for number and period of oscillations and for cumulative depolarization amplitude. Note that the number of locomotor-like oscillations using the hopping pattern is significantly reduced with respect to ReaL*i*stim (*n* = 5; *P* = 0.011).

Figure 5A shows that ReaL*i*stim (period = 1.07 ± 0.10 sec and intensity of stimulation peak = 0.5 Th; top row shows pattern at fast time base) depolarized VRs by 0.22 mV and evoked FL of 53.04 sec duration, with 22 locomotor cycles (period = 2.51 ± 0.55 sec; CV = 0.22). Figure 5B indicates that, on the same in vitro preparation, the EMG pattern obtained during pedaling (period = 1.07 ± 0.11 sec; intensity of stimulation peak = 0.4 Th; top row shows pattern at fast time base) delivered to the same DR induced cumulative depolarization (0.23 mV) with FL (lasting 41.09 sec with 18 locomotor-like oscillations; period = 2.42 ± 0.44 sec; CV = 0.18). Finally, stimulation with a pattern corresponding to the repeated hopping (Fig. 5C; period = 0.58 ± 0.02 and amplitude of stimulation peak = 0.5 Th; top row shows pattern at fast time base) generated a cumulative depolarization (0.20 mV) with short FL (13 oscillations for 28.96 sec; cycle period = 2.41 ± 0.34 sec; CV = 0.14).

FL episodes have been quantified with respect to the number of oscillations (Fig. 5D), cycle period (Fig. 5E) and peak of cumulative depolarization (Fig. 5F), for the three different stimulating protocols delivered to the same five preparations. The ReaL*i*stim appeared to activate the in vitro CPG more efficiently than the EMG pattern recorded during hopping, as shown by the significantly greater number of alternating cycles evoked.

**Figure 6:**
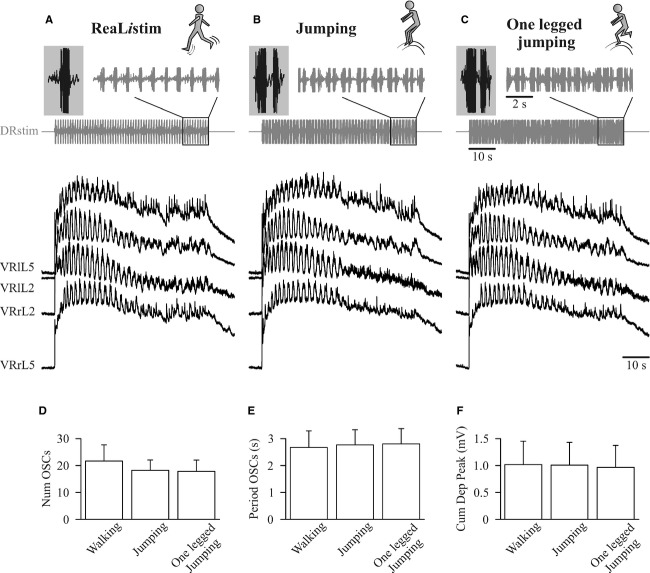
Human EMGs sampled during walking, two legged jumping and one legged jumping provide stimulating waveforms that similarly activate the in vitro CPG. A, ReaL*i*stim is designed from the EMG recordings of the rGM during locomotion (duration 60 sec; top panel shows faster sample trace). The gray box on the left (1 sec width) shows a typical single burst of an EMG during a gait cycle. The stimulating pattern applied to DRrL7 (intensity = 0.1 Th) elicited an epoch of FL from the controlateral L2 and L5 VRs. A similar locomotor-like response was generated by EMGs obtained from rGM muscle (duration = 60 sec; intensity = 0.1 Th) and sampled while the volunteer was jumping on two legs (B). B, top panel on the left shows a single burst, while a sample of the stimulating pattern is illustrated on a faster time base scale. Applying a rGM EMG trace (duration = 60 sec; intensity = 0.1 Th) recorded during one legged jumping generated a comparable episode of FL (C). Top panels in C show one single burst, and 10 sec stimulating pattern (faster time scale). Note that all data depicted in A, B, and C were obtained from the same preparation. Histograms in D, E, and F indicate that no statistical difference appeared in response to the three stimulating protocols, as far as number and period of oscillations, and cumulative depolarization amplitude were concerned (*n* = 6).

We next designed another stimulating pattern that requires the synchronous activation of limbs, like hopping, carried out at a periodicity approximating the one of ReaL*i*stim. To this aim, EMGs of right VM, TA, and GM during both two legged and one legged jumping were sampled. Figure 6A–C compares the EMG records used for ReaL*i*stim (see example of a single burst in the gray box; period of 1.00 ± 0.01 sec), with two legged or one legged jumping. It is noteworthy that despite the EMG similar periodicity, namely of 1.25 ± 0.04 and 1.20 ± 0.03 sec, respectively, bursts for EMGs referred to the two legged or one legged jumping (see examples in the gray boxes of Fig. 6B and C), show the characteristic biphasic component due to, first, contraction of the muscle for the spring phase and, second, the contraction during the return phase.

In Figure 6A, ReaL*i*stim sampled from the rGM induced, at the peak of a cumulative depolarization of 0.49 mV, an episode of 60.58 sec with 30 oscillations, of 2.08 sec period (CV = 0.30). In the same spinal cord, a stimulus of an amplitude approximating that of ReaL*i*stim, sampled from the rGM during jumping, depolarized VRs by 0.51 mV and induced a locomotor episode of 31.591 sec duration and 18 oscillations (period = 1.87 sec and CV = 0.20; Fig. 6B). Similarly, the subsequent delivery to the same DR of EMGs provided of equal intensity and sampled during one legged jumping, determined a depolarization of 0.46 mV and an episode of FL comparable to the ones obtained with the other two patterns of stimulation (duration = 38.30, number of oscillations = 21; period = 1.91 sec, CV = 0.22; Fig. 6C).

The histograms of Figure 6D–F quantify FL episodes evoked by these stimulating patterns (average of six preparations) in terms of the number of oscillations (Fig. 6D), cycle period (Fig. 6E), and peak of cumulative depolarization (Fig. 6F): thus, noisy patterns with a periodicity similar to that of ReaL*i*stim, but sampled during tasks involving the synchronous activation of lower limbs, were equally able to activate the in vitro CPG.

### Stimulating patterns sampled from human EMGs during rhythmic single joint flexions can activate FL

**Figure 7:**
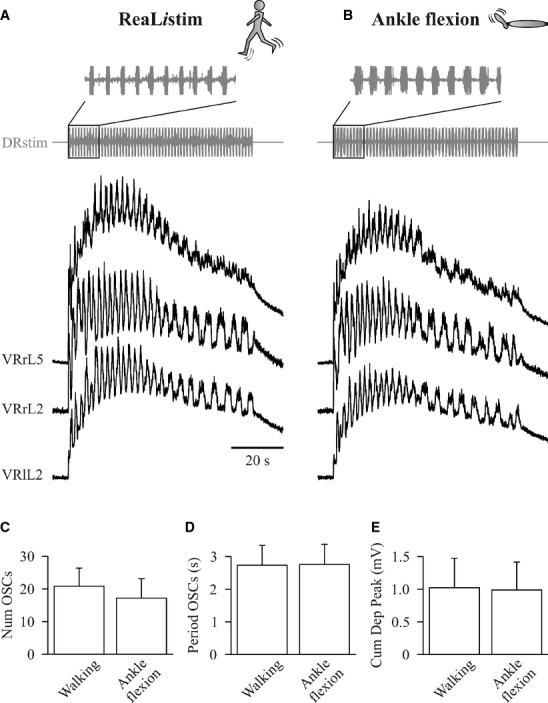
Stimulating patterns obtained from human EMGs from the rhythmic oscillations of ankle joint generate locomotor-like responses similar to the ones induced by ReaL*i*stim. A, ReaL*i*stim (60 sec duration, 0.2 Th intensity), sampled from rGM and delivered to DRrS4, induced a series of alternating oscillations among VRs. At the top, a segment of the trace comprised in the open box is displayed at faster time scale. An analogous response is recorded in B from the same preparation when stimulated with the EMG (60 sec duration, 0.2 Th intensity) captured from rTA during rhythmic oscillations of the ankle joint. A segment of the stimulating pattern (open box) is displayed at the top on a faster time scale. The mean values of pooled data from six experiments are summarized in the histograms below, as for number of oscillations (C), cycle period (D), and peak of cumulative depolarization (E), demonstrating that these two stimulating patterns were similarly effective in inducing an episode of FL with similar characteristics.

We wanted to assess whether phasic EMGs, with the same periodicity as ReaL*i*stim and obtained in the absence of multisegmental movement of lower limbs, were appropriate to generate FL. For this reason, the activity from TA and GM muscles was recorded during rhythmic oscillations of the ankle joint under weight-bearing conditions, with a cycle period (1.09 ± 0.01 sec) approximating that for walking, cycling, and jumping (gray trace in Fig. 7B). Stimulation with a ReaL*i*stim sampled from the rGM induced a depolarization of 1.20 mV with a superimposed episode of 60.37 sec duration with 26 locomotor oscillations of a period of 2.41 sec (CV = 0.38; Fig. 7A). A pattern of similar amplitude to the one of ReaL*i*stim, but obtained from the rTA during repetitive ankle flexions, generated an episode of FL with similar features (cumulative depolarization = 1.14 mV; duration = 58.50 sec; number of oscillations = 23; period of oscillations = 2.67 sec with CV = 0.39; Fig. 7B). Average values obtained from six spinal cords are quantified in terms of number of oscillations, cycle period, and cumulative depolarization in Figure 7D–F.

In conclusion, in the present study, all stimulating patterns obtained from noisy and phasic EMGs, characterized by a periodicity approximating that of ReaL*i*stim, were able to activate the in vitro CPG, regardless of the type of rhythmic task from which they were obtained.

## Discussion

The present study shows that using the EMG records obtained from human leg muscles during normal gait (ReaL*i*stim) and applying them to a single DR of the rat isolated spinal cord was very effective in eliciting a long-lasting series of FL cycles. This novel observation demonstrates that the type of DR stimulus (containing locomotor-related signals) was important rather than its origin (in vitro rat spinal cord or human muscle activity) and that, whether recorded from an in vitro preparation or a healthy volunteer, the rat locomotor networks reacted with similar responses.

### Characteristics of the ReaL*i*stim protocol to activate FL

A protocol of undulatory, noisy stimuli from the human leg EMG during real locomotion (named ReaL*i*stim) activated locomotor-like oscillations in the isolated spinal cord even when the stimulus amplitude was subthreshold to induce a VR reflex. Interestingly, EMG records sampled from flexor or extensor leg muscles were equally effective to evoke FL.

Nevertheless, reconstructing the DR stimulation protocols from the kinematic records of the same human walking activity (Kstim) was not able to induce FL despite collecting stimulus data from either the heel or the metatarsal joint. Thus, the information of simple stimulus alternation contained in the kinematic profile at walking speed could not be sufficient for FL. Likewise, sustained EMG discharge sampled during standing posture or during the execution of isometric squat exercises failed to activate FL. These results are reminiscent of previous data when a broad range of DR stimulation patterns was used to elicit sustained FL in vitro with poor success (Taccola 2011). Thus, we reasoned that the original FL record obtained from the spinal cord in vitro or the ReaL*i*stim obtained from human gait must contain certain properties that confer them the ability to activate the locomotor CPG.

In fact, an epoch of FL analogous for duration and number of oscillations to the one evoked by ReaL*i*stim has been previously observed with FL*i*stim elicited by NMDA + 5-HT (Taccola 2011; Dose and Taccola 2012).

### The importance of noise for electrical stimuli eliciting FL

If the smooth kinematic stimulus pattern as well as the high-frequency firing during isometric contraction were unable to produce FL, we suspected that the intrinsic variability of the stimulating traces used in this study was a crucial element for an optimal CPG activation. This notion is in accordance with previous observations that the efficacy of stimulation with noisy waves was lost when the stimulating pattern was smoothed (Taccola 2011). This result reaffirms the importance of variability for spinal CPG function (Ziegler et al. 2010; Lee et al. 2011), in line with error-based motor learning paradigms (Huang et al. 2011).

As EMG records cannot provide detailed information on the nature of these electrical signals, we have indicated such traces as “noisy”, based on the sole macroscopic observation of the ragged baseline. In line with this point of view, the ReaL*i*stim protocol apparently possesses the same level of noise as FL*i*stim. However, intrinsic variability of FL*i*stim mainly corresponds to the firing profile of motoneurons within the same pool during FL (Berg et al. 2007), while for the EMG of real locomotion in a volunteer, additional nonlinear sources of variability need to be considered. For instance, stiffness, viscoelastic properties of muscles, coupling among limb segments and biomechanical constrains, anticipatory adjustments from supraspinal centers, and reflex responses to external perturbations are likely contributors to the noise within in vivo EMGs (Thrasher et al. 2011). Other forms of variability in human EMG signals are attributable to specific muscle pennation and fiber composition (Johnson et al. 1973) or to the proximity of EMG electrodes to the muscle innervation (De Luca 1997). These sources of EMG variability seemed negligible in activating the CPG because EMGs from either different leg muscles or the same muscle in different recording sessions appeared to have the same ability to elicit FL.

Thus, baseline noise associated with a certain waveform with main frequency within the real or fictive locomotion rhythms was the crucial requirement for activating the locomotor network in vitro. Another possibility is that the noise sampled from the EMG or from the FL contains distinctive information that codifies the state of network activity and the type of motor task undertaken. In support of this notion is the report that in the cerebral cortex the noise fluctuations in neuronal network output rely on the frequency of sensory stimuli in a state-dependent manner (White et al. 2012).

Comparison of power spectra of noisy waveforms shows low-frequency domain components, which seem to distinguish ReaL*i*stim from the isometric contraction protocol (ineffective in recruiting the CPG). Future work will be necessary to explore whether low-frequency components are sufficient per se in activating FL or if they also need the high-frequency background that characterizes all noisy stimulating patterns from EMG recordings.

### Phasic EMGs of frequency similar to ReaL*i*stim equally activated the CPG regardless of their task specificity

The first part of the study reported the optimal locomotor-like response evoked by stimulating waves, sampled from limb muscles during the execution of rhythmic tasks, such as walking and cycling, that both involve an alternated pattern. On the other hand, EMGs obtained during synchronous rhythmic activation of lower limbs (e.g., hopping) appeared less effective. This difference may be attributed to certain intrinsic characteristics of the various EMGs. In fact, although the stimulating pattern was always recorded from one single leg, the EMG of even one muscle during alternated activation of lower limbs (running or pedaling) may contain distinct information for the activation of the in vitro CPG. The same does not seem to occur, though, with the EMG sampled during the execution of tasks that require the synchronous activation of limbs, like hopping (Bizzi et al. 2008). Examples of task specificity in network output have already been reported and attributed to the modulation provided by the differential afferent feedback that characterizes different motor tasks (Brooke et al. 1992).

In the present experiments, comparing the stimulating effectiveness of EMGs from hopping or walking is misleading, as their cycle period was clearly different. Despite repeated attempts, volunteers failed to hop and to walk at identical frequency. Hence, to compare ReaL*i*stim with the one obtained with EMGs from two legged synchronous movements, we recorded EMGs from different muscles during jumping with both legs at the same main frequency as gait. Stimulation with EMGs obtained from one legged jumping was also tested to evaluate the influence of the proprioceptive information coming from the rhythmic movement of the controlateral limb (Savin et al. 2010). In fact, it has been demonstrated the existence of a movement-related afferent feedback originating from network interaction of inputs arising from the two limbs (McIlroy et al. 1992; Peper and Carson 1999) and this modulation seems to be accounted by presynaptic inhibitory mechanisms (Stein 1995). In particular, passive movement of one limb can drive phase and frequency of the controlateral one (Gunkel 1962; deGuzman and Kelso 1991).

In our experiments, stimulation with EMGs obtained from both two legged and one legged jumping did not appear to be statistically different from ReaL*i*stim in inducing FL. Thus, most noisy patterns recorded during limb movement, as long as they possess a main frequency similar to the one of ReaL*i*stim, appeared to be equally effective in activating the CPG, regardless of the motor task under which they were taken.

Furthermore, to confirm the scarce task specificity of EMG traces to induce a FL, we stimulated the isolated spinal cord with noisy and phasic patterns of a main frequency equal to that of locomotor patterns, but recorded during a nonpropulsive action of lower limbs, such as rhythmic flexions of the ankle in weight-bearing conditions and observed little task specificity of the stimulating patterns.

### Future perspectives

Although stimulation of a dorsal or sacral afferent with a noisy wave represents the most powerful tool to electrically generate the locomotor rhythm in the isolated neonatal rat spinal cord (Taccola 2011), to date there is no clinical experience in the use of a similar protocol for epidural or peripheral stimulation.

Solving the complexity of the undulatory asynchronous stimulus used in our experiments may facilitate the introduction of new parameters for clinical electrostimulators. For this purpose, the isolated spinal cord, thanks to its well defined dorsal input and ventral motor output, represents a very useful model to assess the degree of recruitment of locomotor network through afferent electrical inputs.
